# Atypical Membrane Topology and Heteromeric Function of *Drosophila* Odorant Receptors In Vivo

**DOI:** 10.1371/journal.pbio.0040020

**Published:** 2006-01-17

**Authors:** Richard Benton, Silke Sachse, Stephen W Michnick, Leslie B Vosshall

**Affiliations:** **1**Laboratory of Neurogenetics and Behavior, The Rockefeller University, New York, New York, United States of America; **2**Département de Biochimie, Université de Montréal, Montréal, Québec, Canada; Howard Hughes Medical Institute, California Institute of TechnologyUnited States of America

## Abstract

*Drosophila* olfactory sensory neurons (OSNs) each express two odorant receptors (ORs): a divergent member of the OR family and the highly conserved, broadly expressed receptor OR83b. OR83b is essential for olfaction in vivo and enhances OR function in vitro, but the molecular mechanism by which it acts is unknown. Here we demonstrate that OR83b heterodimerizes with conventional ORs early in the endomembrane system in OSNs, couples these complexes to the conserved ciliary trafficking pathway, and is essential to maintain the OR/OR83b complex within the sensory cilia, where odor signal transduction occurs. The OR/OR83b complex is necessary and sufficient to promote functional reconstitution of odor-evoked signaling in sensory neurons that normally respond only to carbon dioxide. Unexpectedly, unlike all known vertebrate and nematode chemosensory receptors, we find that *Drosophila* ORs and OR83b adopt a novel membrane topology with their N-termini and the most conserved loops in the cytoplasm. These loops mediate direct association of ORs with OR83b. Our results reveal that OR83b is a universal and integral part of the functional OR in *Drosophila*. This atypical heteromeric and topological design appears to be an insect-specific solution for odor recognition, making the OR/OR83b complex an attractive target for the development of highly selective insect repellents to disrupt olfactory-mediated host-seeking behaviors of insect disease vectors.

## Abstract 2

This study reveals a novel membrane topology for olfactory receptors in *Drosophila* and details the molecular mechanisms of receptor localization at the sensory cilia.

## Introduction

Animals have the remarkable ability to detect and discriminate thousands of chemically distinct odors. Mammals and insects have solved this complex sensory perception problem in strikingly similar ways. In both phylogenetic classes, odors are detected by large families of highly divergent odorant receptors (ORs) [1]. Individual olfactory sensory neurons (OSNs) express only one, or rarely a few, OR genes [2–4], and OR proteins are exposed to the environment on the surface of the ciliated endings of OSN dendrites [5–7]. ORs appear to be the major determinant of the odor-response profile of OSNs and are activated or inhibited by overlapping subsets of odor stimuli [8–12]. The axons of OSNs expressing the same OR converge to form glomeruli within the olfactory bulb in mammals or the antennal lobe in insects, where they synapse with second-order neurons [3,13,14]. Together, these observations have led to a model in which the identity of an odor is encoded, in both mammals and insects, by the combination of ORs that recognize it, and therefore in distinct spatial patterns of glomerular activity in the brain [15,16].

While the olfactory neuroanatomy and physiology of these animals are similar, mammalian and insect OR families are not obviously related, although both have a predicted seven-transmembrane (TM) domain structure [1]. Mammalian ORs are members of the Class A rhodopsin-like G protein–coupled receptor (GPCR) superfamily and signal through the G alpha subunit G_olf_ [17,18]. The structural similarity of the insect proteins has led to the widespread assumption that insect ORs represent a divergent class of GPCR [19–21], but almost nothing is known about the signal transduction cascade they activate. Furthermore, while each mammalian OSN expresses a single OR that determines its functional specificity, insect OSNs express a conventional ligand-binding OR together with OR83b, a highly conserved member of the insect OR family [22–25]. Flies lacking OR83b display severe defects in behavioral and electrophysiological responses to many different odors, indicating that OR83b is likely to act in conjunction with conventional ORs that are expressed in smaller subpopulations of OSNs. Consistent with this hypothesis, the conventional receptors OR22a/b and OR43b are highly degraded in *Or83b* mutant neurons with trace quantities of these proteins detected only in the cell body [22].

In contrast to the absolute requirement for OR83b in vivo, insect ORs can produce small, but ligand-specific odor responses when expressed without OR83b in heterologous cells [26,27]. However, the efficiency of odor responsivity is greatly enhanced by co-expression of OR83b [28,29], and this may be due to the stabilization of ORs by OR83b [28]. Bioluminescence-resonance–energy-transfer experiments indicate that OR–OR homomeric and OR–OR83b heteromeric complexes form in heterologous cells, but evidence of the functionality of either complex is lacking [29]. Thus, while these in vitro findings support the hypothesis that OR83b can act with the conventional ORs, its precise role in vivo remains a mystery.

Remarkably little is known about the molecular and cellular properties of ORs in OSNs, and a number of mechanisms could account for the *Or83b* loss-of-function phenotype and the enhancement promoted by OR83b in vitro. OR83b could function as a chaperone in intracellular organelles to promote the folding, assembly, or stability of ORs, or their exit from the endoplasmic reticulum (ER). OR83b could be essential for transporting and depositing ORs in the specialized sensory cilia at the tip of OSN dendrites. Finally, OR83b could be a co-receptor that remains in a complex with ORs in the sensory compartment and participates in anchoring or stabilizing of ORs in the ciliary membrane, odor binding, olfactory signaling, or a combination of these.

In this work, we analyze the subcellular localization, physical interactions, and function of ORs in vivo to address the precise molecular role of OR83b. We present compelling evidence that the functional insect odorant-receptor is a heteromeric OR–OR83b complex whose formation is critical for the localization to and maintenance in the sensory cilia. This heteromeric receptor is sufficient for the full reconstitution of an odor response in ectopic sensory neurons that do not normally express ORs. We make the surprising discovery that insect ORs adopt a membrane topology distinct from that of GPCRs, with their N-termini located intracellularly. These proteins associate via conserved loops that were previously thought to be extracellular. These results define the molecular nature of the functional odorant receptor in insects as a co-receptor complex of a novel family of TM proteins and indicate that, despite the anatomical and functional parallels in the mammalian and insect olfactory systems, insects have evolved a completely different molecular solution to detect odors.

## Results

### OR83b Is Required for OR Trafficking

The major olfactory organ of *Drosophila* is the third segment of the antenna, a cuticle-covered appendage that contains approximately 1,200 OSNs (Figure 1A, left panel) [30]. The surface of this organ is covered with porous sensory hairs, or sensilla, which are of three major morphological classes (basiconic, coeloconic, and trichoid), and house the dendrites of between one and four OSNs (Figure 1A, middle panel) [31]. OSN dendrites comprise a proximal inner segment and a distal-ciliated outer segment (Figure 1A, right panel). There are 62 ORs in *Drosophila,* and 37 of these are expressed in specific subpopulations of antennal OSNs that display characteristic odor response profiles [3,9,19–21,32–34]. OR83b is estimated to be co-expressed with these ORs in 70%–80% of antennal OSNs [12,22,35].

**Figure 1:**
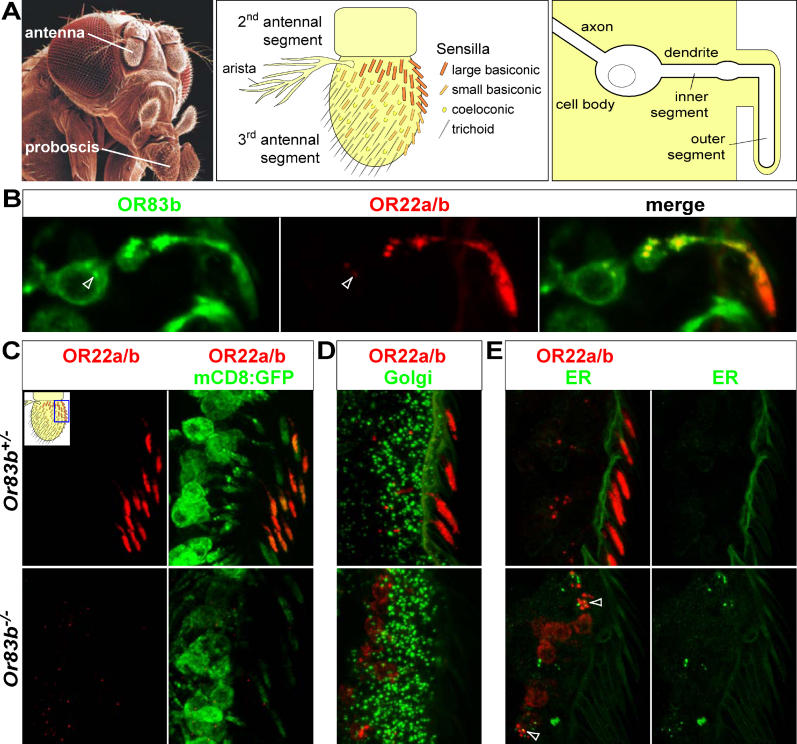
OR83b Is Essential for OR Membrane Trafficking (A) The *Drosophila* olfactory system. Left panel: false-colored scanning electron micrograph image of a *Drosophila* head illustrating the major olfactory (antenna) and gustatory (proboscis) organs (photo: Jürgen Berger, Max Planck Institute for Developmental Biology, Tübingen, Germany). Center panel: schematic of olfactory sensilla distribution on the third antennal segment. Right panel: schematic of OSN anatomy. (B) Immunostaining for OR83b (green) and OR22a/b (red) in antennal sections of a wild-type (*yw*) animal. Arrowheads mark enrichment of perinuclear OR83b that co-localize with the trace amount of OR22a/b present in the cell body. (C) Immunostaining for OR22a/b (red) and mCD8:GFP (α-GFP, green) in antennal sections of control heterozygous (*Or83b^1^/+*, top row) and homozygous (*Or83b^1^/Or83b^2^*, bottom row) *Or83b* null-mutant animals. The position of the field of view, in this and subsequent figures, is illustrated by the blue square in the antennal schematic (inset in upper left panel). Images of control and mutant samples were taken at identical confocal settings to permit comparison of signal intensities. Therefore, levels of OR22a/b are lower in (C) than in (D and E), in which confocal settings have been adjusted to permit visualization of OR22a/b in *Or83b* mutants. (D) Immunostaining for OR22a/b (red) and Golgi (α-Golgi, green) in antennal sections of control heterozygous (top panel) and homozygous (bottom panel) *Or83b* null-mutant animals. (E) Immunostaining for OR22a/b (red) and ER (α-KDEL, green) in antennal sections of heterozygous control (top panel) and homozygous (bottom panel) *Or83b* null-mutant animals. Mislocalized OR22a/b and KDEL accumulations overlap in the mutant (arrowheads in lower left panel). Autofluorescence of the antennal cuticle is visible in the green channel at the base of the sensilla. In (D and E), confocal settings of the red channel were adjusted to permit clear visualization of the weaker OR22a/b signal in *Or83b* mutants.

Conventional ORs, such as OR22a/b, are concentrated in the outer dendritic segment, where they co-localize with OR83b (Figure 1B) [22]. OR83b is also abundant in the cell body in a perinuclear rim and is enriched in regions that co-localize precisely with the small fraction of OR22a/b detected in the cell body (Figure 1B, arrowheads). In the absence of OR83b, OR22a/b is highly unstable and detected only at very low levels in the neuronal cell bodies (Figure 1C) [22]. Despite these dramatic effects on OR localization, OSN morphology and membrane organization appear normal in *Or83b* mutants when visualized using the membrane marker mCD8:GFP (Figure 1C).

We next investigated the distribution of mislocalized OR22a/b in *Or83b* mutants with respect to intracellular organelles. The *Drosophila* Golgi apparatus has a punctate distribution (Figure 1D, top panel), which is unaffected in *Or83b* mutants. Mislocalized OR22a/b does not co-localize with this Golgi marker (Figure 1D, bottom panel). We observed a similar lack of co-localization of OR22a/b with endosomal and lysosomal compartments labeled by a GFP:RAB7 fusion protein (unpublished data), suggesting that OR trafficking is impaired prior to arrival at the Golgi.

The ER was visualized using a KDEL antibody, which recognizes the major retention signal for soluble ER proteins. In wild-type OSNs, α-KDEL displays extremely faint perinuclear staining (Figure 1E, top panels). In contrast, in *Or83b* mutants, brightly stained KDEL accumulations are observed (Figure 1E, bottom panels). These accumulations are specific to neuronal cell bodies as determined by examining KDEL staining in antennae in which *Or83b* mutant neurons are also labeled with mCD8:GFP (unpublished data). In contrast to the fully penetrant defects in OR localization, these accumulations are detected in only a small fraction of OSNs (<20%), which suggests that they may be a secondary consequence of the failure in OR trafficking. Mislocalized OR22a/b partially overlaps with these accumulations when they occur in *Or22a/b* neurons, consistent with at least a fraction of ORs being retained in the ER in the absence of OR83b (Figure 1E, arrowheads). These changes in ER organization could have indirect effects on the forward transport of other membrane proteins, which may account for the reduced levels of mCD8:GFP in *Or83b* mutant dendrites (Figure 1C, bottom panel).

### OR83b Is Continuously Required for OR Localization

To assess when OR83b is required for OR localization, we performed rescue experiments of *Or83b* mutants using the TARGET (temporal and regional gene expression targeting) system to achieve temporal control of OR83b expression, specifically in *Or22a/b* neurons [36]. This technique combines cell-type specific induction of a *UAS-Or83b* transgene by an *Or22a-Gal4* driver line with temporal regulation through use of a ubiquitously expressed temperature-sensitive *Gal80 (Gal80ts)* transgene. At the permissive temperature (18 °C), the GAL80 protein is active and represses induction of OR83b expression by inhibiting GAL4 (Figure 2A, top row). At the restrictive temperature (29 °C), GAL80 is inactivated, permitting expression of OR83b in OR22a/b neurons (Figure 2A, bottom row).

**Figure 2:**
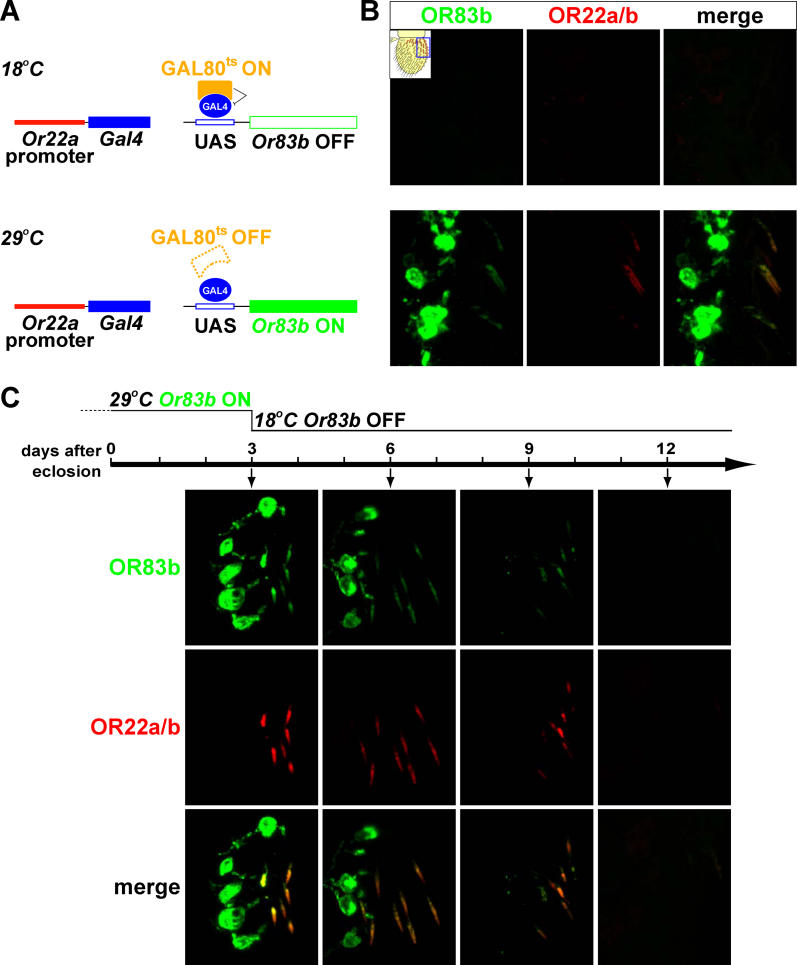
OR83b Is Required to Maintain OR Localization in Adults and Has No Essential Developmental Function (A) Schematic of the TARGET system. (B) Immunostaining for OR83b (green) and OR22a/b (red) in *Or83b* null-mutant flies rescued using a *UAS-Or83b* transgene under the control of the TARGET system (*Or22a-Gal4,tubP-Gal80ts/tubP-Gal80ts;UAS-Or83b,Or83b^1^/tubP-Gal80ts,Or83b^2^*). Three copies of the *tubP-Gal80ts* transgene were used to ensure efficient suppression of GAL4 activity by GAL80 at 18 °C. Flies were cultured and aged as adults for 10 d at 18 °C and then incubated for a further 2 d either at 18 °C (top row) or at 29 °C (bottom row) to induce late expression of *Or83b*. (C) Immunostaining for OR83b (green) and OR22a/b (red) in flies [genotype as in (B)] that were cultured and aged as adults for 3 d at 29 °C, transferred to 18 °C to switch off *Or83b* expression, and stained at the time points indicated.

In the first experiment, we cultured flies at 18 °C, collected adults, and aged these for 10 d at 18 °C. We then split these animals into two groups and incubated them for a further 2 d at either 18 °C or 29 °C before fixing and staining. Flies that had been maintained continuously at 18 °C do not express OR83b, and OR22a/b is absent from the cilia (Figure 2B, top row). In contrast, OR83b expression is robustly induced in flies that had been transferred to 29 °C and OR22a/b is localized correctly to the sensory compartment (Figure 2B, bottom row). This result indicates that late expression of OR83b is sufficient to promote OR22a/b localization and rules out a developmental role for OR83b in cilia morphogenesis or OR trafficking.

In a second experiment, we cultured flies and aged adults for 3 d at 29 °C, transferred them to 18 °C to switch off expression of OR83b, and fixed these animals immediately for 3, 6, or 9 d later (Figure 2C). We observe a progressive decline in OR83b expression levels in the cell body, although protein perdures in the sensory cilia for several days after levels in the cell body become undetectable. OR22a/b shows an essentially parallel decline in the cilia, and we never detect OR22a/b in the sensory compartment in the absence of OR83b. We conclude that OR83b is essential to maintain OR localization and stability.

### OR83b-Dependent and -Independent Trafficking Pathways in the Antenna

To examine the spatial requirements for OR localization, we first ectopically expressed one OR, OR43a, bearing an N-terminal GFP tag, throughout the antenna using *Or83b-Gal4*. GFP:OR43a is functional (see below) and in wild-type tissue localizes to cilia in all *Or83b*-expressing neurons (Figure 3A, left panels). In contrast, in *Or83b* mutant antennae, GFP:OR43a is delocalized and destabilized, with only a weak signal detected in OSN cell bodies (Figure 3A, right panel). OR83b is therefore essential for OR localization in all neurons in which it is detectably expressed, and no cell-type specific factors appear to be required for trafficking of individual ORs.

**Figure 3:**
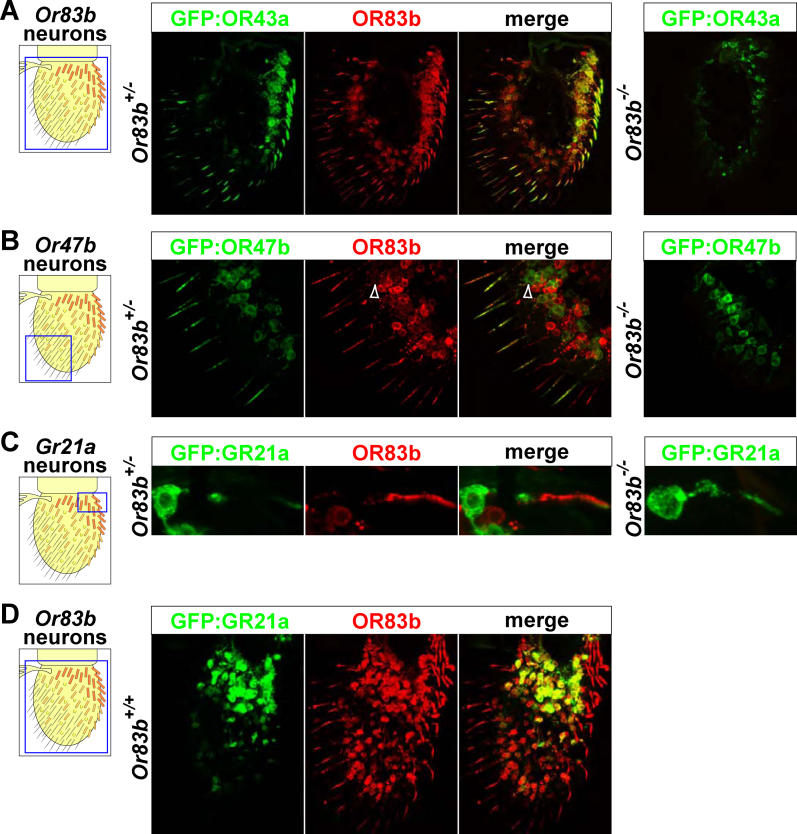
Spatial Requirements for OR83b in OR Localization (A) Immunostaining of GFP:OR43a (α-GFP, green) expressed in all *Or83b* neurons and OR83b (red) in antennal sections of control heterozygous (*Or83b-Gal4/UAS-GFP:Or43a;Or83b^1^/+*, left panels) and homozygous (*Or83b-Gal4/UAS-GFP:Or43a;Or83b^1^/Or83b^2^,* right panel) *Or83b* null-mutant animals. (B) Immunostaining for GFP:OR47b (α-GFP, green) expressed in *Or47b* neurons and OR83b (red) in antennal sections of control heterozygous (*Or47b-Gal4/UAS-GFP:Or47b;Or83b^1^/+*, left panels) and homozygous (*Or47b-Gal4/UAS-GFP:Or47b;Or83b^1^/Or83b^2^,* right panel) *Or83b* null-mutant animals. Arrowheads illustrate low level of OR83b expression in *Or47b* neurons. (C) Immunostaining for GFP:GR21a (α-GFP, green) expressed in *Gr21a* neurons and OR83b (red) in antennal sections of control heterozygous (*Gr21a-Gal4/UAS-GFP:Gr21a;Or83b^1^/+*, left panel) and homozygous (*Gr21a-Gal4/UAS-GFP:Gr21a;Or83b^1^/Or83b^2^,* right panel) *Or83b* null-mutant animals. The OR83b signal in this sensillum reflects expression in the non-*Gr21a*-expressing neurons ab1A, ab1B, and ab1D, whose olfactory responses are *Or83b*-dependent [22,32]. (D) Immunostaining for GFP:GR21a (α-GFP, green) expressed in all *Or83b* neurons and OR83b (red) in antennal sections of wild-type animals (*Or83b-Gal4/+;UAS-GFP:Gr21a/+)*.

*Or83b* mutant adult flies retain some olfactory function [22], and, given the heterogeneous expression of OR83b in the antenna (Figure 3A), we wondered whether this reflects the function of OR83b-independent ORs. We investigated this in *Or47b* neurons, which are situated in the lateral-distal region of the antenna where OR83b is expressed at the lowest levels. GFP:OR47b was selectively expressed in *Or47b* neurons, and these were stained for OR83b and GFP. Although these neurons express only extremely low levels of OR83b (Figure 3B, left panels, arrowheads), the localization of GFP:OR47b to the cilia remains dependent upon OR83b (Figure 3B, compare left and right panels). This suggests that OR83b is likely to function with all ORs, regardless of its expression level.

OR genes represent an expanded lineage of the ancestral chemosensory family of gustatory receptor (GR) genes [37]. GR genes are primarily expressed in gustatory neurons, but at least three are detected in antennal sensory neurons, including *Gr21a,* which is expressed in the ab1C neurons that respond specifically to carbon dioxide (CO_2_) [32,38,39]. The CO_2_ response of ab1C neurons is independent of *Or83b* [22]. Consistent with this physiological phenotype, no OR83b is present in *Gr21a* neurons (Figure 3C, left panel), and the ciliary localization of GFP:GR21a is unaffected in *Or83b* mutants (Figure 3C, compare left and right panels). However, GFP:GR21a fails to localize to cilia when misexpressed in *Or83b* neurons (Figure 3D), suggesting that factors selectively present in *Gr21a* neurons are essential for its localization. Thus, although OR and GR protein families are related, ORs must have evolved distinct molecular properties that confer their absolute dependence upon OR83b.

### OR-Independent Localization of OR83b to OSN Ciliated Dendrites

We tested whether there is a reciprocal requirement for conventional ORs for the ciliary accumulation of OR83b by examining OR83b localization in the absence of OR22a/b. *Or22a/b* mutant neurons display no odor-evoked potentials to any odorant tested, indicating that no other ORs are likely to be expressed in these neurons [7]. To distinguish the dendrites of *Or22a/b* neurons from those of *Or85b* neurons, which share the same sensillum [9], we expressed an N-terminal GFP-tagged version of OR83b specifically in *Or22a/b* neurons. GFP:OR83b is functional, as assayed by rescue of odor-evoked behavior in *Or83b* mutant larvae (M. Louis, RB, and LBV, unpublished data), and its distribution in the cell body and dendrite of wild-type *Or22a/b* neurons is identical to endogenous OR83b (Figure 4, top row). This localization is unchanged *Or22a/b* in mutant neurons (Figure 4, bottom row), but levels of GFP:OR83b are reduced in both the cell body and the cilia. This indicates that OR83b is partly destabilized in the absence of a conventional OR, but that it can localize to sensory cilia independently of ORs.

**Figure 4:**
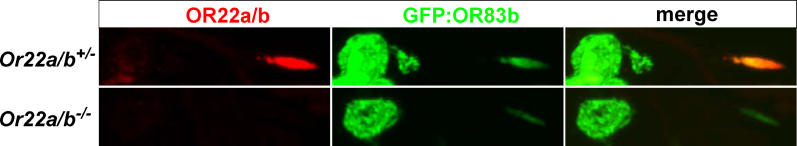
OR-Independent Localization of OR83b to OSN Ciliated Dendrites Immunostaining for OR22a/b (red) and GFP:OR83b (α-GFP, green) in antennal sections of control heterozygous (*Or22a/b^Δhalo^/+;Or22a-Gal4/UAS-GFP:Or83b*, top panels) and homozygous (*Or22a/b^Δhalo^/Or22a/b^Δhalo^;Or22a-Gal4/UAS-GFP:Or83b,* bottom panels) *Or22a/b* null-mutant animals. Images of control and mutant samples were taken at identical confocal settings to permit comparison of signal intensities.

### OR83b Is Necessary and Sufficient to Mediate OR Localization to Ciliated Dendrites in Other Sensory Neurons

To ask whether OR83b is sufficient to promote OR localization, we ectopically expressed GFP:OR43a with and without OR83b in sensory neurons that do not normally express ORs. When GFP:OR43a is expressed alone in *Gr21a* neurons, the protein fails to localize to the sensory compartment and is detected only in the cell body and inner dendritic segment (Figure 5A, top row). In contrast, OR83b localizes to cilia, albeit extremely weakly, when expressed alone in these neurons (Figure 5A, middle row, arrowhead). However, when these proteins are co-expressed, both GFP:OR43a and OR83b display clear localization to the ciliated outer segment (Figure 5A, bottom row). Similar results are obtained by misexpression of ORs and OR83b in the ciliated mechanosensory neurons in the second antennal segment, which mediate the perception of sound [40] (Figure 5B). We conclude that OR83b is the only protein required to couple ORs to a transport pathway common to ciliated neurons.

**Figure 5:**
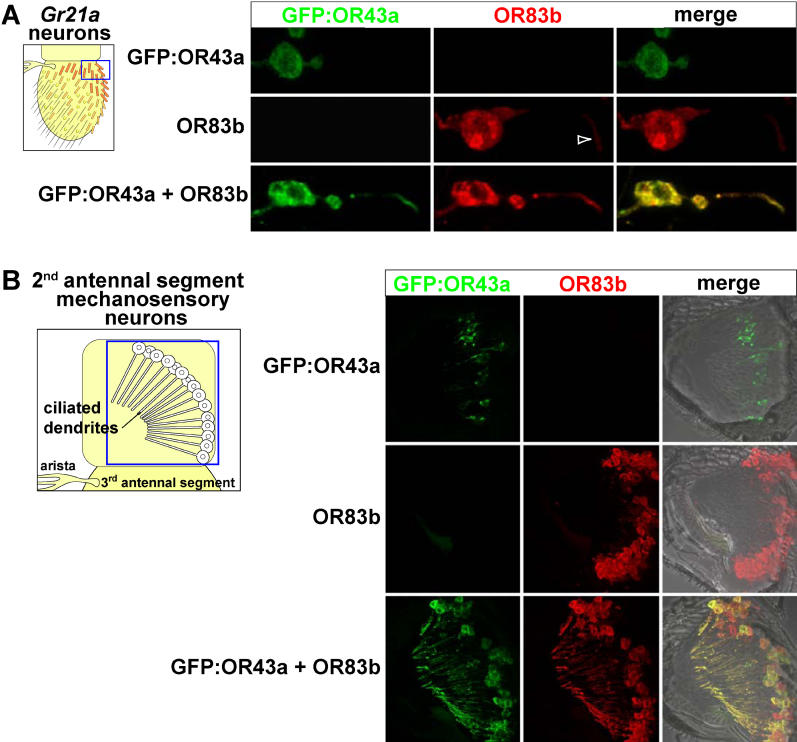
OR83b Is Necessary and Sufficient to Mediate OR Localization to Ciliated Dendrites in Other Sensory Neurons (A) Immunostaining for GFP:OR43a (α-GFP, green) and OR83b (red) when misexpressed singly or in combination in *Gr21a* neurons. These animals are homozygous mutant for *Or83b*, which allows visualization of protein distributions specifically in *Gr21a* neurons. The arrowhead marks very weak localization of OR83b to cilia when expressed alone. Genotypes: Top row: *Gr21a-Gal4/UAS-GFP:Or43a;Or83b^1^/Or83b^2^*. Middle row: *Gr21a-Gal4/+;UAS-Or83b,Or83b^1^/Or83b^2^*. Bottom row: *Gr21a-Gal4/UAS-GFP:Or43a;UAS-Or83b,Or83b^1^/Or83b^2^*. (B) Immunostaining for GFP:OR43a (α-GFP, green) and OR83b (red) when misexpressed singly or in combination in second antennal segment mechanosensory neurons using the ciliated cell-specific *oseg2-Gal4* driver. OR83b shows extremely weak localization to cilia that is not visible under these imaging conditions. Merged images are overlaid on a bright-field image to visualize tissue morphology. Genotypes: Top row: *oseg2-Gal4/UAS-GFP:Or43a*. Middle row: *oseg2-Gal4/+;UAS-Or83b/+*. Bottom row: *oseg2-Gal4/UAS-GFP:Or43a;UAS-Or83b/+*.

### OR/OR83b Reconstitutes a Functional OR in *Gr21a* Neurons

We investigated whether ORs and OR83b form a functional odorant receptor in *Gr21a* cilia, by ectopically expressing GFP:OR43a and OR83b in these neurons along with the calcium-sensitive fluorescent reporter G-CaMP [12,41]. Odor-evoked activity was measured as changes in intracellular calcium concentration in the axon termini of *Gr21a* neurons in the V glomerulus of the antennal lobe [38,39].

In flies expressing only G-CaMP, robust responses are observed, as expected, to CO_2_, but not to cyclohexanol, a known OR43a ligand [26,42] (Figure 6A, left column). When GFP:OR43a and OR83b are co-expressed in these neurons, significant calcium increases in response to cyclohexanol stimulation are now observed (Figure 6A, right column). Significant responses of GFP:OR43a/OR83b in *Gr21a* neurons are also observed with cyclohexanone, hexanol, benzaldehyde, isoamyl acetate, and geranyl acetate but not octanol, linalool, or caproic acid (Figure 6B). These results are consistent with previous reports of the ligand specificity of *Or43a* [9,12,26,42]. Thus, OR83b is the only factor required with OR43a to reconstitute a functional OR that is capable of recognizing ligands and stimulating neuronal signaling.

**Figure 6:**
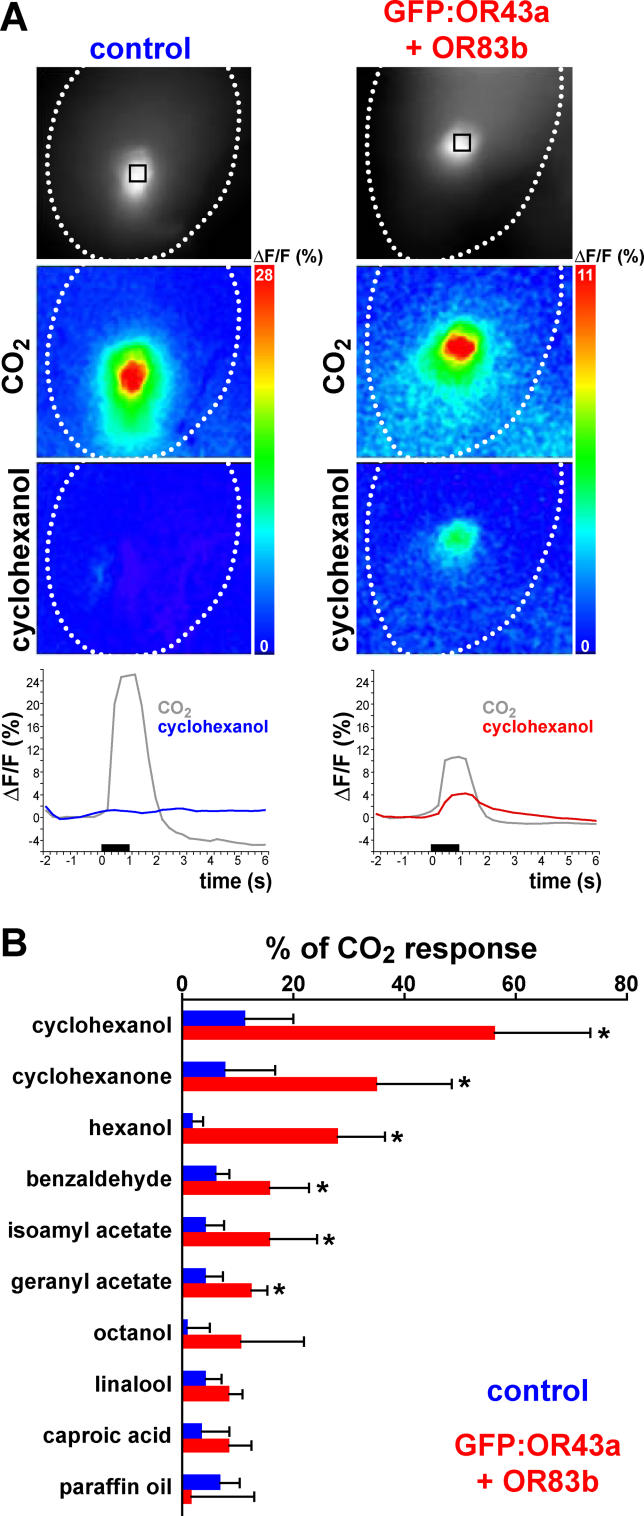
Expression of ORs and OR83b Reconstitutes a Functional OR in *Gr21a* Neurons (A) Representative stimulus-evoked calcium signals recorded from the axon terminals of *Gr21a* neurons in the antennal lobe V glomerulus of a control animal (*UAS-G-CaMP/UAS-G-CaMP;Gr21a-Gal4/Gr21a-Gal4*; left column) and an animal misexpressing OR83b and GFP:OR43a in *Gr21a* neurons (*UAS-G-CaMP/+;Gr21a-Gal4/UAS-GFP:Or43a;UAS-G-CaMP/UAS-Or83b;* right column). Top row: intrinsic G-CaMP fluorescence of the V glomerulus. Dotted lines mark the antennal lobe border, and the black squares mark the area of the V glomerulus evaluated for stimulus-evoked changes in fluorescence. Middle row: false-color–coded images during stimulation with CO_2_ (5%) or cyclohexanol (10^−2^) represent ΔF/F (%) according to the scales on the right panel. Bottom row: time traces of stimulus-evoked signals of the V glomerulus. Black bars indicate odor stimulation time. The diminished responses to CO_2_ in animals expressing GFP:OR43a/OR83b may reflect competition between the resident and ectopic receptors in engaging the ciliary trafficking pathway or downstream signaling components. (B) Normalized odor-evoked calcium responses of control (blue) and GFP:OR43a/OR83b-misexpressing (red) animals [genotypes as in (A)] expressed as a percentage of the CO_2_ response in each genotype. GFP:OR43a/OR83b-misexpressing animals show stronger responses than control animals for the odor stimuli (all at 10^−2^) marked with an asterisk (*p* < 0.05; two-tailed unpaired *t*-test; *n =* 4 animals per genotype and stimulus). Chemical Abstracts Service (CAS) registry numbers: cyclohexanol (108–93–0), cyclohexanone (108–94–1), hexanol (111–27–3), benzaldehyde (100–52–7), isoamyl acetate (123–92–2), geranyl acetate (105–87–3), octanol (111–87–5), linalool (126–91–0), caproic acid (142–62–1).

### ORs and OR83b Form Heteromeric Complexes in the Sensory Cilia of OSNs

We next asked whether this functional odorant receptor is composed of a complex of OR and OR83b proteins that is present in vivo at the site of odor detection (Figure 7). Previous in vitro efforts suggested that ORs can form both homomers and heteromers with OR83b, but no evidence was offered that these complexes are functional [29]. To investigate the existence of OR/OR83b complexes in OSNs, we employed the protein-fragment complementation assay (PCA), using a yellow fluorescent protein (YFP) reporter [43]. In this technique, complementary N-terminal and C-terminal fragments of YFP [YFP(1) and YFP(2)] are fused to two proteins suspected to interact. The two halves of YFP are not fluorescent alone and do not associate spontaneously, but the physical interaction of the proteins to which they are fused brings the YFP fragments into proximity where they can fold into an active form. The YFP fluorescent signal output therefore not only provides direct evidence for the existence of protein complexes in vivo but also information on their subcellular distribution [44]. YFP fragments were fused to ORs via a flexible ten-amino-acid linker that has a fully extended length of about 40 Å. With this linker, complex formation can therefore be detected between directly interacting proteins or those that are within 80 Å of each other, which is approximately twice the diameter of the helical bundle of the rhodopsin monomer [45,46].

**Figure 7:**
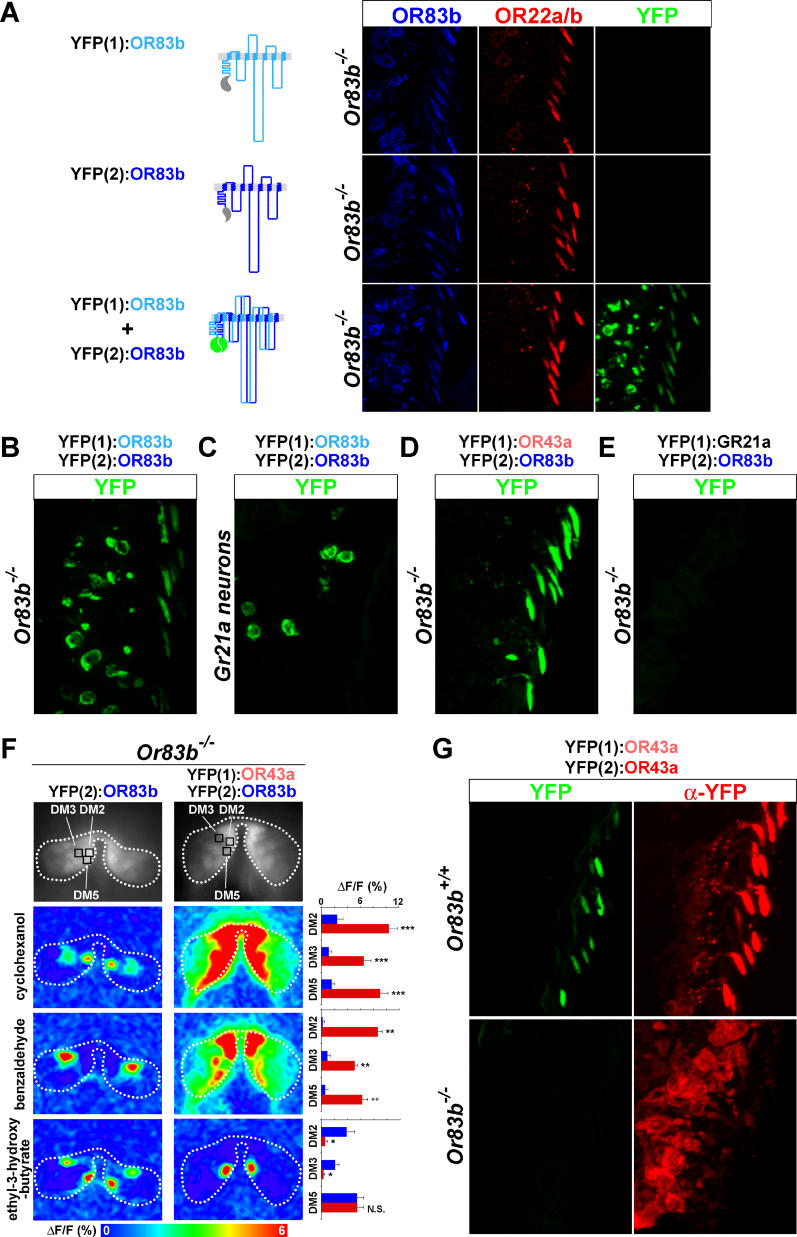
In Vivo Formation and Distribution of OR/OR83b Complexes (A) Immunostaining for OR83b (blue), OR22a/b (red), and intrinsic YFP fluorescence (green) in antennal sections of *Or83b* null-mutant animals expressing YFP fragment:OR83b fusions, singly or in combination, as illustrated in the snake plots on the left. We note that the snake plots in this and subsequent figures are generated by computational analysis of OR sequences and the exact number and precise placement of the TM domains has not been experimentally verified. Genotypes: *Or83b-Gal4/UAS-YFP(1):Or83b;Or83b^1^/Or83b^2^* (top row); *Or83b-Gal4/+;UAS YFP(2):Or83b,Or83b^1^/Or83b^2^* (middle row); *Or83b-Gal4/UAS-YFP(1):Or83b;UAS-YFP(2):Or83b,Or83b^1^/Or83b^2^* (bottom row). (B–E) Intrinsic YFP fluorescence (green) in antennal sections of animals expressing the indicated combinations of complementary YFP fragment fusions with these genotypes: (B) *Or83b-Gal4/UAS-YFP(1):Or83b;UAS-YFP(2):Or83b,Or83b^1^/Or83b^2^* (C) *Gr21a-Gal4/UAS-YFP(1):Or83b;UAS-YFP(2):Or83b/+* (D) *Or83b-Gal4/UAS-YFP(1):Or43a;UAS-YFP(2):Or83b,Or83b^1^/Or83b^2^* (E) *Or83b-Gal4/UAS-YFP(1):Gr21a;UAS-YFP(2):Or83b,Or83b^1^/Or83b^2^* (F) Left column: representative stimulus-evoked calcium signals recorded from the axon terminals of *Or83b* neurons in the antennal lobe of an *Or83b* mutant animal expressing YFP(2):OR83b alone (*UAS-G-CaMP/UAS-G-CaMP;Or83b-Gal4/+;UAS-YFP(2):Or83b,Or83b^1^/Or83b^2^*) or YFP(1):OR43a and YFP(2):OR83b (*UAS-G-CaMP/UAS-G-CaMP;Or83b-Gal4/UAS-YFP(1):Or43a;UAS-YFP(2):Or83b,Or83b^1^/Or83b^2^*) as indicated. Top row: intrinsic G-CaMP fluorescence in glomeruli innervated by *Or83b* neurons. Dotted lines mark the antennal lobe border and the black squares mark the area of the three selected glomeruli (DM2, DM3, and DM5) evaluated for stimulus-evoked changes in fluorescence. Below are false-color-–coded images during stimulation with two characterized OR43a ligands (cyclohexanol and benzaldehyde, both at 10^−3^ dilution) and a control odor that does not activate OR43a (ethyl-3-hydroxybutyrate, 10^−5^ dilution) representing ΔF/F (%) according to the scale at the bottom. Right column: quantification of odor-evoked calcium responses in the three indicated glomeruli of animals expressing YFP(2):OR83b alone (blue) or YFP(1):OR43a (red), and YFP(2):OR83b YFP(1):OR43a/YFP(2):OR83b-expressing animals show stronger responses for the known OR43a ligand stimuli (** *p* < 0.01; *** *p* < 0.001). DM2 and DM3 glomeruli show reduced responses to the control odor (* *p* < 0.05) similar to the effects of ectopic OR expression on the endogenous CO_2_ responses of *Gr21a* neurons (Figure 6), while DM5 activity does not differ significantly between genotypes (marked N.S. on the bar graph). Significance was assessed with a two-tailed unpaired *t*-test; *n* = 4 animals per genotype and stimulus. CAS Registry Numbers: cyclohexanol (108–93–0), benzaldehyde (100–52–7), ethyl-3-hydroxybutyrate (54058–41–4). (G) Intrinsic YFP fluorescence (green) and immunostaining for YFP (red) in antennal sections of animals expressing YFP(1):OR43a and YFP(2):OR43a in control heterozygous (*Or83b-GAL4/UAS-YFP(1):Or43a;UAS-YFP(2):Or43a,Or83b^1^/+* [top panel]) and homozygous (*Or83b-GAL4/UAS-YFP(1):Or43a;UAS-YFP(2):Or43a,Or83b^1^/Or83b^2^* [bottom panel]) *Or83b* null-mutant animals.

Transgenic constructs encoding YFP(1):OR83b and YFP(2):OR83b fusion proteins were first expressed individually in *Or83b* mutant neurons (Figure 7A, top and middle rows). Immunostaining reveals that YFP(1):OR83b and YFP(2):OR83b localize normally and are functional as they rescue the ciliated localization of OR22a/b, but neither protein alone displays detectable YFP fluorescence. When co-expressed, however, a strong YFP fluorescence signal is detected in these neurons, providing evidence for homomeric complex formation by OR83b (Figure 7A, bottom row, and 7B). Two lines of evidence indicate that this homomerization reflects an intrinsic property of OR83b and does not depend upon the presence of conventional ORs: first, abundant YFP fluorescence is detected within both the cell body and sensory dendrites of these neurons even though OR22a/b protein is concentrated in the sensory compartment (Figure 7A, bottom row). Second, YFP fluorescence is detected when these fusion proteins are expressed in *Gr21a* neurons, where no OR is expressed (Figure 7C). We note that formation of homomers by OR83b does not preclude functional interaction with conventional ORs, as these complexes retain the ability to promote OR22a/b localization (Figure 7A, bottom row).

To assess heteromeric interactions between ORs and OR83b, YFP(1)- and YFP(2)-tagged versions of OR43a were expressed with complementary YFP(1/2):OR83b fusions in *Or83b* mutant neurons. Both combinations of these fusion proteins produce a robust fluorescent signal in the sensory cilia, with discrete puncta of fluorescence also observed around the nucleus in the cell body and in the inner dendritic segment (Figure 7D). Thus, OR83b forms heteromeric complexes with conventional ORs in vivo, and these are concentrated at the site of odor detection.

We investigated whether these heteromeric complexes are functional by expressing these fusion proteins along with G-CaMP in *Or83b* mutant neurons and assessing odor-evoked calcium release at OSN axon terminals in the antennal lobe (Figure 7F). In control animals that express YFP(2):OR83b alone, sparse glomerular activation patterns in response to two known OR43a ligands (cyclohexanol and benzaldehyde) are observed (Figure 7F, left column). These are similar to activity patterns seen in wild-type animals [12], indicating that YFP(2):OR83b is able to rescue odor-evoked responses of endogenous ORs. Co-expression of YFP(1):OR43a with YFP(2):OR83b results in odor-evoked calcium responses across broad domains of the antennal lobe (Figure 7F, right column), and quantification reveals highly significant increases in individual glomerular response properties (Figure 7F, far right column). These ectopic glomerular responses are due to the activity of YFP(1):OR43a in these neurons because a control odor that does not activate OR43a (ethyl-3-hydroxybutyrate) gives similar glomerular activation patterns in both genotypes (Figure 7F, bottom row). Thus, the YFP(1):OR43a/YFP(2):OR83b heteromer is functional for odor-evoked neuronal signaling.

As a control for the specificity of these complexes, we generated YFP fragment fusions to GR21a. These localize throughout the cell body in *Or83b* neurons and the inner segment of the dendrite, although not in the outer segment, as shown for GFP:GR21a (Figure 3D; unpublished data). When placed in complementary combinations with either YFP-tagged OR43a or OR83b, only an extremely faint fluorescent signal is detected in the cell body (Figure 7E). Similar results are obtained when YFP(1):GR21a and YFP(2):OR83b are co-expressed in *Gr21a* neurons (unpublished data). This suggests that the fluorescence observed between different combinations of OR83b and OR43a results from the formation of specific receptor complexes, rather than the mere presence of complementing YFP-fusion proteins within the same membrane.

We also observe relatively weak fluorescence in sensory cilia when YFP(1):OR43a and YFP(2):OR43a fusions are co-expressed in wild-type *Or83b* neurons (Figure 7G, top panel), suggesting that OR43a might homomultimerize. To ask whether this fluorescence signal reflects indirect interactions between OR43a molecules within multimeric OR43a/OR83b complexes, we expressed these fusion proteins in *Or83b* mutant neurons (Figure 7G, bottom panel). Although the YFP(1/2):OR43a fusion proteins are detected in the cell body by immunostaining (Figure 7G, bottom panel, right side), no intrinsic YFP fluorescence signal is detected (Figure 7G, bottom panel, left side). These observations suggest that, in contrast to in vitro results [29], conventional ORs are unable to associate directly in homomeric complexes in OSNs without OR83b and reinforce the specificity of the formation of OR83b homomers and OR43a/OR83b heteromers.

### ORs and OR83b Adopt a Novel Membrane Topology

To define the regions that mediate the specific association of ORs with OR83b, we initiated a structure/function analysis of these receptors. This was initially constrained by the lack of knowledge of their membrane topology and structure, as *Drosophila* ORs were identified bioinformatically by algorithms that searched for novel proteins with multiple TM domains [19–21,47]. Although these reports all proposed a seven-TM domain structure for the identified sequences, there was no consensus on the placement of these TM segments. By analogy to vertebrate and *Caenorhabditis elegans (C. elegans)* ORs, the predicted heptahelical structure of *Drosophila* ORs has led to the general acceptance that these proteins represent members of the GPCR family, despite the fact that the insect proteins show no significant sequence similarity to any known GPCR (unpublished data). Indeed, phylogenetic analysis suggests that insect ORs define a distinct family that is no more related to mouse ORs than these are to ion channels (Figure 8A). Given this apparent novelty in the primary structure of *Drosophila* ORs*,* we analyzed their membrane topology using the HMMTOP algorithm [48] and compared this with a representative sample of mouse ORs. Surprisingly, although the majority of sequences are predicted to contain seven TM domains for both organisms (Figure 8B), the membrane orientation predictions of these families are distinct (Figure 8C). Mouse ORs are predicted to have an extracellular N-terminus, which is consistent with the known structure of the GPCR superfamily. In contrast, *Drosophila* ORs are predicted to have an intracellular N-terminus (Figure 8C). Similar predictions are obtained for the ORs with two independent algorithms, TMHMM Server version 2.0 [49] and TMpred [50]), and in analysis of the GR protein family (unpublished data).

**Figure 8:**
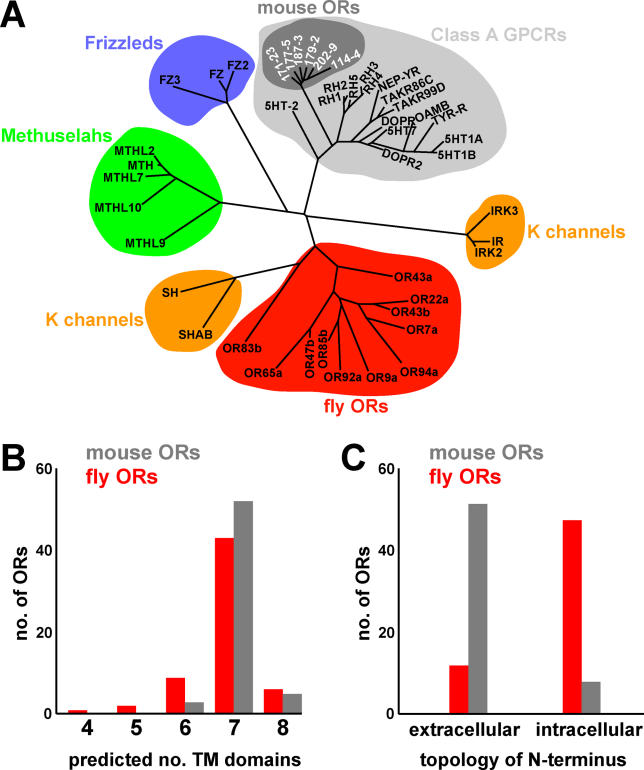
Bioinformatic Analysis Defines *Drosophila* ORs As a Novel Family of TM Proteins (A) Unrooted neighbor-joining tree of selected *Drosophila* ORs [37], Class A GPCRs [84]*,* Methuselah family receptors [85], Frizzled receptors [86], potassium channels, and mouse ORs [87]. Sequences were aligned in ClustalX with 1000 bootstrap iterations. (B) TM domain predictions of *Drosophila* ORs (*n* = 61) and a representative subset of mouse ORs (*n* = 61) by the HMMTOP version 2.0 algorithm, including all those ORs depicted in (A). (C) Membrane-insertion orientation predictions of *Drosophila* and mouse ORs (same sets as in (B)) by HMMTOP version 2.0. All mouse ORs mispredicted to have an intracellular N-terminus (8/8) and most *Drosophila* ORs predicted to have an extracellular N-terminus (8/12) are not predicted to have seven TM domains, suggesting that this algorithm may have difficulty in analyzing these particular sequences.

To obtain experimental evidence that TM1 of *Drosophila* ORs inserts into the membrane with the N-terminus intracellular, we first used the β-galactosidase β-gal fusion technique. This method takes advantage of the observation that β-gal is enzymatically active when present in the cytosolic compartment but not in extracytosolic compartments (luminal or extracellular) [51]. By fusing β-gal to the C-terminus of a TM domain and assessing enzymatic activity, the cellular location of the enzyme and hence orientation of insertion of the TM domain can be determined (Figure 9A, top row). This assay does not require that the resulting fusion proteins are trafficked to the cell surface but merely assays the orientation of protein insertion in the ER.

**Figure 9:**
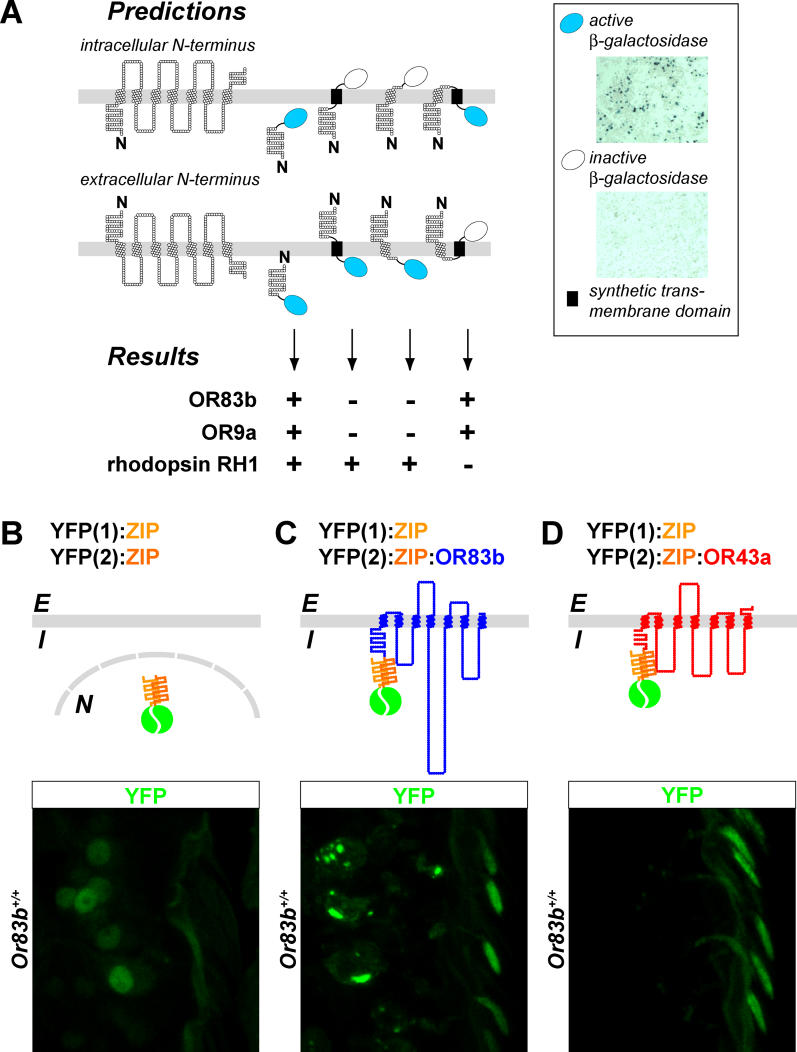
The N-Terminus of *Drosophila* ORs Is Intracellular (A) Determination of OR N-terminus membrane insertion orientation by the β-gal fusion technique in S2 cells. Top left bars: schematic of fusion constructs and predictions of β-gal activity for proteins with an intracellular or extracellular N-terminus. Top right panels: sample field of view of S2 cells expressing OR83b N-term: β-gal (top panel) and OR83b N-term:artificial TM domain: β-gal (bottom panel) stained with X-gal to reveal active and inactive β-gal. Bottom table: active (+) and inactive (−) β-gal in the indicated OR83b, OR9a, and *Drosophila* RH1 fusion proteins. (B–D) Intrinsic YFP fluorescence (green) in antennal sections of animals expressing the indicated combinations of complementary YFP fragment and ZIP dimerization domain fusions with these genotypes: (B*) Or83b-Gal4/+;UAS-YFP(1):zip/UAS-YFP(2):zip* (C) *Or83b-Gal4/UAS-YFP(2):zip:Or83b;UAS-YFP(1):zip/+* (D) *Or83b-Gal4/+;UAS-YFP(1):zip/UAS-YFP(2):zip:Or43a*

We generated constructs encoding either the N-terminal domain alone or the N-terminal domain and TM1 of OR83b fused at their C-termini to β-gal. As a control, we generated constructs in which a synthetic TM domain was placed between the fragments of OR83b and β-gal, which are predicted to give opposite results to the corresponding direct fusions to the enzyme (Figure 9A, top row). These constructs were expressed in cultured *Drosophila* S2 cells, and β-gal activity was assessed by X-gal staining. β-gal was scored as active if 10%–20% of cells were blue, which corresponded to the transfection efficiency in these experiments, and inactive if <1% cells were blue. The results are consistent with an intracellular localization of the OR83b N-terminus (Figure 9A, bottom row). Identical results were obtained with fusion constructs of the conventional OR, OR9a. In contrast, equivalent fusions with N-terminal fragments of the class A GPCR, *Drosophila* rhodopsin RH1, give results consistent with the extracellular location of the RH1 N-terminus. These results support the computational prediction that the N-terminus of *Drosophila* ORs is intracellular.

We wished to determine whether OR N-termini reside intracellularly in the context of the full-length proteins in OSNs, and we therefore developed a novel method to probe protein topology in vivo based upon the YFP PCA. We generated transgenes encoding cytosolic topology-sensor proteins that comprise YFP fragments fused to a leucine zipper dimerization domain (referred to here as ZIP) (YFP(1):ZIP, YFP(2):ZIP) and corresponding OR83b fusion proteins bearing YFP fragments and the same leucine zipper sequence at their N-termini (YFP(1):ZIP:OR83b, YFP(2):ZIP:OR83b). YFP(1/2):ZIP:OR83b fusions are functional as assessed by rescue of OR22a/b localization (unpublished data).

When the complementary YFP(1/2):ZIP cytosolic sensors are expressed in OSNs, the ZIP domains promote their association and we detect YFP fluorescence concentrated in the nucleus (Figure 9B), probably reflecting the tendency of small cytoplasmic proteins to translocate to this compartment. When these sensors are co-expressed with the complementary YFP(1/2):ZIP:OR83b proteins, fluorescence is observed in the puncta in the cell body and throughout sensory cilia (Figure 9C). As the reconstitution of YFP fluorescence can occur only if both YFP fragments are present on the same side of the membrane, these results demonstrate unambiguously that the OR83b N-terminus is located in the cytoplasm. Similar results are obtained with combinations of YFP(1/2):ZIP and YFP(1/2):ZIP:OR43a fusions (Figure 9D). We also note that the heteromeric OR43a/OR83b complexes observed in OSNs by the PCA (Figure 7D) could only have been observed if the N-terminal YFP tags are topologically equivalent. Thus, both OR83b and conventional OR N-termini are located intracellularly in vivo, and the association of ORs with cytosolic topology sensors causes these sensor proteins to be relocalized to ciliated dendrites.

We generated equivalent extracytosolic topology sensors bearing the signal sequence from mammalian calreticulin at their N-termini that targets these proteins to the secretory pathway (SS:YFP(1):ZIP, SS:YFP(2):ZIP) [52]). In OSNs expressing these sensors, we detect α YFP immunoreactivity in perinuclear membranes consistent with their targeting to the ER lumen (unpublished data). When co-expressed in complementary combinations with either YFP(1/2):ZIP:OR83b or YFP(1/2):ZIP:OR43a, we do not observe reconstitution of intrinsic YFP fluorescence (unpublished data), which is consistent with these extracytosolic sensors being on the opposite side of the membrane of the OR N-termini. Interpretation of this result must be tempered, however, by the fact that the combination of SS:YFP(1):ZIP and SS:YFP(2):ZIP fails to fluoresce either in the intracellular sorting pathway or extracellularly (unpublished data).

To examine OR topology beyond the location of the N-terminus, we performed OR83b antibody epitope-staining experiments, which probe topology by comparing antibody access in permeabilized and non-permeabilized conditions. OSN dendrites proved to be inaccessible to labeling without compromising cell permeability. We find that the larval salivary gland, a secretory tissue that is easily accessible to whole-mount staining, appears to support cell-surface expression of ectopically expressed GFP:OR83b, with the intrinsic GFP fluorescent signal detected in membranes along the cell boundaries and within cytoplasmic vesicles (Figure 10A and 10B) similar to the pan-membrane localization of this protein in OSNs (Figure 3). To determine whether GFP:OR83b is on the cell surface, we stained these cells with antibodies against GFP and the OR83b α-EC2 antibody, which recognizes an epitope within the computationally predicted second extracellular loop, either in the presence or absence of detergent. Under permeabilized conditions, both antibodies detect the entire pool of protein (Figure 10B, top row). In contrast, when the cells are unpermeabilized, the GFP antibody fails to show staining, while the OR83b α-EC2 antibody labels the cell boundary, representing the fraction of protein within the plasma membrane (Figure 10B, middle row). This staining is specific, as it is not observed in unpermeabilized salivary glands that do not express GFP:OR83b (Figure 10B, bottom row). These observations demonstrate that GFP:OR83b is present on the surface of these cells, confirm that the N-terminal GFP tag is located intracellularly, and indicate that the EC2 epitope is exposed on the extracellular face of the membrane.

**Figure 10:**
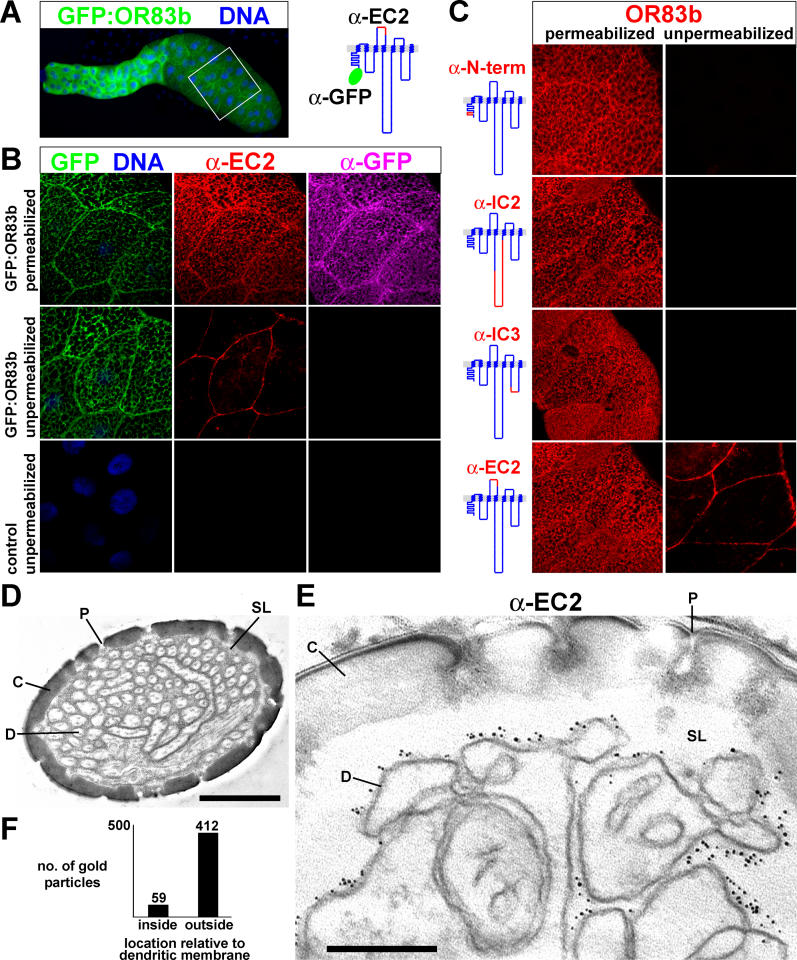
Probing OR83b Topology by Antibody Epitope Staining (A) Left panel: whole-mount view of a third instar larval salivary gland expressing GFP:OR83b (green) counterstained with DAPI (blue) to visualize the cell nuclei. Genotype in this and subsequent panels: *AB1-Gal4/+;UAS-GFP:Or83b/+*. The white box marks the approximate field of view of this tissue shown in all subsequent panels. Right bar graphic: snake plot of OR83b showing the predicted topological location of the N-terminal GFP epitope and the OR83b α-EC2 antibody epitope. (B) Immunostaining of GFP:OR83b (intrinsic fluorescence in green) in larval salivary gland cells with α-EC2 (red) and α-GFP (purple) when permeabilized (0.25% Triton X-100 detergent, top row) or unpermeabilized (no detergent, middle row). The cell membrane staining of OR83b α-EC2 under unpermeabilized conditions is not detected in control salivary glands (*AB1-Gal4/+*) (bottom). Images are single confocal sections of cells in a plane through or just above the cell nuclei (visualized with DAPI staining, blue). (C) Salivary glands expressing GFP:OR83b (*AB1-Gal4/+;UAS-GFP:Or83b/+*) were stained with antibodies against the epitopes, illustrated in red in the snake plots on the left, under permeabilized or unpermeabilized conditions. For clarity, only the red channel is shown. None of the antibodies stain control salivary glands under permeabilized conditions (unpublished data). (D) Horizontal section of an antennal sensillum viewed by conventional EM reveals cross-sections of dendritic membranes (scale bar = 1 μm). C, cuticle; P, pore; D, dendrite; SL, sensillum lymph. (E) ImmunoEM on a horizontal section of an antennal sensillum using OR83b α-EC2 and a secondary antibody conjugated to 5 nm colloidal gold reveals distribution of the EC2 epitope on the extracellular face of the dendritic membranes (scale bar = 200 nm). (F) Quantification of gold particle distribution scored from four sections obtained in two independent experiments.

We next used antibodies against the OR83b N-terminus and epitopes within the computationally predicted second and third intracellular loops (IC2 and IC3) and performed a similar set of experiments. Unlike α-EC2, these three antibodies display staining only when the cells are permeabilized (Figure 10C), supporting an intracellular location of these epitopes. Identical results are obtained when using untagged OR83b (unpublished data), indicating that the GFP tag does not influence protein topology.

Because topology mapping by epitope access with detergents has inherent limitations (e.g., [53]), we performed immunoelectron microscopy (immunoEM) in cross sections of wild-type sensilla to ask where the α-EC2 epitope lies relative to the dendritic membrane. Horizontal sections reveal multiple dendritic branches within the sensillum lymph by conventional EM (Figure 10D). For immunoEM, we prepared ultrathin plastic sections and stained these with the OR83b α-EC2 antibody and a secondary antibody conjugated to 5 nm gold. To permit antibody access, more gentle fixation procedures are used and these somewhat distort dendritic membrane morphology (Figure 10E). Nevertheless, gold particles are detected specifically along the dendritic membranes, consistent with the expected membrane localization of OR83b (Figure 10E). Moreover, these gold particles show a striking bias in their distribution, with 87.5% of gold particles (*n* = 471) found outside the boundaries of the ciliary membranes (Figure 10F). Together with the immunofluorescence analysis in the salivary gland, this result indicates an extracellular location for the EC2 epitope.

Together, these data support the bioinformatic prediction that the EC2 epitope of OR83b is extracellular, while the N-terminus and the IC2 and IC3 epitopes are intracellular. Although we have obtained multiple lines of evidence for the topology of the OR N-terminus, we note that the exact number and precise placement of TM segments in the *Drosophila* ORs remain to be proven. One prediction of the model presented in the snake plots illustrated in the figures is that the C-terminus is extracellular. Unfortunately, we have not been able to test this experimentally, because, unlike the N-terminal GFP tag, fusion of GFP or the smaller Myc tag to the OR83b C-terminus destroys protein function (unpublished data).

### ORs and OR83b Associate via Conserved Cytoplasmic C-Terminal Domains

To examine the domains that mediate OR/OR83b association, we used this new topology model to design a chimeric receptor [OR83b(1–170):OR43a(159–376)] with a breakpoint in EC2 such that the protein comprises predicted TM1–TM3 of OR83b and TM4–TM7 of OR43a. This chimera localizes to cilia in wild-type antennae, but fails to localize in *Or83b* mutants (Figure 11A). In *Gr21a* neurons, the chimera localizes to ciliated dendrites only when OR83b is co-expressed (Figure 11A). This chimera therefore displays the localization properties of OR43a, suggesting that the C-terminal region of OR43a is sufficient to couple to OR83b-dependent transport to olfactory cilia.

**Figure 11:**
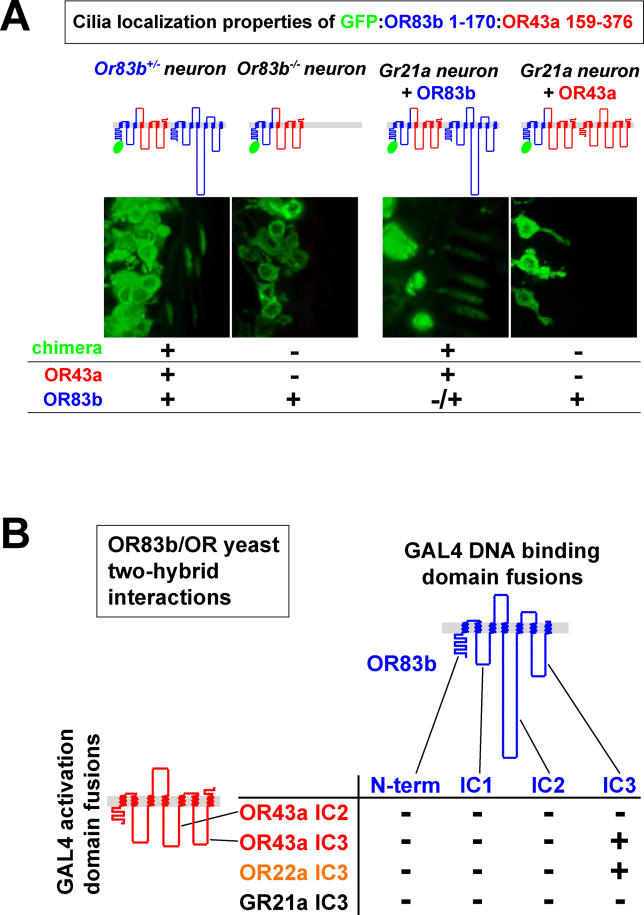
OR83b and ORs Associate via Conserved C-Terminal Domains (A) Top rows: Immunostaining of the GFP:OR83b(1–170):OR43a(159–376) chimera (α-GFP, green) in antennal sections of animals of the following genotypes (left to right): *Or83b^+/−^* neuron [*Or83b-Gal4/UAS-GFP:Or83b(1–170):Or43a(159–376);Or83b^1^/+*]; *Or83b^−/−^* neuron [*Or83b-Gal4/UAS-GFP:Or83b(1–170):Or43a(159–376);Or83b^1^/Or83b^2^*]; *Gr21a* neuron + OR83b [*Gr21a-Gal4/UAS-GFP:Or83b(1–170):Or43a(159–376);UAS-Or83b/+*]; *Gr21a* neuron + OR43a [*Gr21a-Gal4/UAS-GFP:Or83b(1–170):Or43a(159–376);UAS-Rho:Or43a/+*]. Bottom table: summary of localization (+) or no localization (−) to cilia of the chimera and, for comparison, OR83b and OR43a. (B) Interactions between OR83b and OR cytoplasmic domains detected by the yeast two-hybrid assay by observation of growth (+) or no growth (−) of yeast co-transformed with the indicated bait/prey combinations on media selecting for expression of *HIS3* and *ADE2* reporters.

OR protein sequences are extremely divergent but show the strongest homology within this C-terminal region. Given its functional dependence on OR83b, we asked whether any of the computationally predicted cytoplasmic loops within this fragment of OR43a (IC2, IC3) physically interact with any cytoplasmic regions of OR83b (N-term, IC1, IC2, IC3) in a yeast two-hybrid assay. Although this technique analyzes OR interactions without the structural information that might be provided by OR TM domains, this approach has been successfully used to define cytoplasmic associations of many types of polytopic membrane protein (e.g., [54]). We observe interactions between IC3 of OR43a and IC3 of OR83b but not any other combination (Figure 11B). OR83b IC3 also interacts with an equivalent region of OR22a, but not of GR21a, demonstrating that this is a conserved interaction interface specific to the OR family (Figure 11B).

These experiments defining OR/OR83b interactions also provide further evidence that conventional ORs adopt the same topology as OR83b. First, the membrane insertion orientation of the OR83b:OR43a chimera is determined by the N-terminus of OR83b, but this fusion protein retains the localization properties of OR43a (Figure 11A). Second, we observe direct physical interactions between loops of OR83b and ORs that are predicted to be topologically equivalent (Figure 11B).

## Discussion

Our results define *Drosophila* OR83b and ORs as a novel family of TM proteins with sequence and membrane topology that is distinct from mammalian GPCR-family ORs. We show that OR83b associates with ORs through conserved cytoplasmic loops previously believed to be extracellular and demonstrate that ORs and OR83b form heteromeric complexes within OSNs. These complexes form early in the membrane-trafficking pathways but persist and concentrate in the sensory cilia. We show an essential role for OR83b in targeting and maintaining these complexes within the ciliary membranes at the site of odorant signal transduction. These results define OR83b as an integral part of the functional odorant receptor in insects. Furthermore, despite the striking similarities in the anatomy and physiology of mammalian and insect olfactory systems, they reveal important distinctions in the molecular nature of the odorant receptor in these organisms.

### OR83b and OR Localization

The role of ORs in translating the odorous environment into neuronal activity depends critically on their localization to the surface of the ciliated sensory endings of OSN dendrites. How ORs navigate from their site of synthesis in the ER to these specialized sensory compartments is poorly characterized but has long been suspected to depend upon olfactory-specific cofactors because of the difficulty in functionally expressing these proteins in heterologous cells [55]. Our data show that in insects an OR protein has been adapted to subserve a new cellular function: to traffic structurally similar ligand-binding ORs to olfactory cilia.

Our observation that OR83b can localize to chemosensory cilia in the absence of associated ORs rules out the possibility that only the heteromeric OR/OR83b complex is transport-competent. Instead, our data indicate that OR83b itself can associate with the transport pathway in OSNs and functions to link ORs to this transport machinery. Because OR83b can promote OR localization to cilia in mechanosensory neurons, it likely couples to a general ciliary transport pathway, without the requirement for additional OSN-specific cofactors.

Our analysis of the temporal requirement of OR83b also reveals an essential role for OR83b in maintaining ORs within this sensory compartment, as we never detect OR22a/b in cilia in the absence of OR83b. Together with our observation of the persistence of OR/OR83b heteromeric complexes in the sensory compartment, these results strongly suggest that OR83b is an integral and stable component of the insect OR complex necessary for both proper localization and stability of the conventional ORs in dendrites, rather than a transient chaperone that shuttles and deposits monomeric ORs in the ciliary membrane.

While *Drosophila* ORs do not contain any known protein motifs, the strongest homology within members of this family spans predicted TM6 and TM7, suggesting that this region might mediate a function common to all ORs. Consistent with this conservation, we find that the loop linking these predicted TM domains (IC3) forms at least part of the interaction interface between ORs and OR83b. In OR83b, this region is almost fully conserved between insect orthologues, and this may explain why OR83b orthologues from diverse insects can functionally substitute for OR83b in *Drosophila* [24]. Comparison of OR83b with other ORs reveals the presence of a 70-amino-acid insertion in IC2 that is unique to OR83b. Our data suggest this loop is located in the cytoplasm, and we speculate that the insertion links OR83b to the intracellular transport machinery.

Most mammalian ORs fail to reach the cell surface when expressed in heterologous cells but are largely trapped in aggregates in the ER and are eventually degraded [56–58]. This is remarkably similar to the fate of *Drosophila* ORs in *Or83b* mutants. OR trafficking in mammals appears to have been solved differently from insects. Screens for olfactory-specific genes that facilitate OR localization have identified a number of single TM domain accessory factors that associate with but are structurally unrelated to the ORs: REEP, RTP1, and RTP2 for mouse ORs and MHC class 1b proteins for mouse V2R pheromone receptors [59,60]. A single TM domain protein, ODR-4, has also been shown to be required for OR trafficking in *C. elegans* but, unlike OR83b, ODR-4 is not present in sensory cilia [61].

Heterodimerization of structurally related chemosensory receptors is important for mammalian taste perception, but this modulates the ligand-binding properties of these receptors rather than their subcellular localization [62–64]. Thus, the requirement for a universal co-receptor for chemoreception appears to be unique to the *Drosophila* OR family, and this relies on the remarkable property of OR83b to couple both to the conserved ciliary trafficking machinery and probably to all 61 members of the highly divergent OR family.

### Odor Recognition and Olfactory Signaling by a Heteromeric OR/OR83b Complex

The presence of OR/OR83b complexes in the sensory compartment raises the possibility that OR83b has additional functions in olfactory perception. This is difficult to address in vivo because of the essential requirement for OR83b in OR localization. However, the diversity in odor response profiles of different classes of OR83b-expressing neurons makes it unlikely that OR83b itself recognizes ligands. This has been confirmed experimentally in *Or22a/b* mutant neurons, which do not respond to odors even though we demonstrate that OR83b is present in these sensory cilia [7]. In vitro studies have revealed that some ectopically expressed ORs in heterologous cells are capable of recognizing odors in the absence of OR83b, albeit with low efficiency [26,27]. The odor-response profiles of these ORs is similar to that observed in vivo, which suggests that OR83b is unlikely to influence OR ligand specificity but could facilitate efficient ligand-receptor interactions by, for example, maintaining the conformation of ORs within the ciliary membrane.

Although the site of ligand interaction in insect ORs and GRs is unknown, a naturally occurring single amino acid polymorphism (A218T) in GR5a influences the sensitivity of this receptor to trehalose [65–67]. Previously, this residue was thought to lie in the second intracellular loop, where it was proposed to affect coupling of GR5a to G proteins. In the topology model for insect chemosensory receptors proposed here, this residue is predicted to be extracellular and may instead influence ligand binding.

The molecular components of primary olfactory signal transduction pathways in insects are unknown. However, in vivo misexpression experiments indicate that ORs can function in other OR-expressing OSNs [9]. This suggests that ORs converge on a common signaling cascade in *Or83b* neurons, and it is attractive to suggest that OR83b might form part of this common pathway. The capacity of OR83b to couple to downstream signal transduction components is supported by the observation that OSNs expressing OR83b but lacking conventional ORs show spontaneous activity while *Or83b* mutant OSNs do not [7,22,68]. Our demonstration that OR/OR83b complexes can function in *Gr21a* neurons suggests that OR83b may not define a signal transduction pathway that is unique to OR-expressing sensory neurons, but rather that the divergent OR and GR chemosensory receptor families can couple to a common cascade.

The structural distinction between insect and mammalian ORs begs the question of whether G proteins are involved in insect chemosensory signaling. In both mammals and *C. elegans,* loss-of-function genetics provides strong support for a role of G proteins in olfactory signal transduction [18,69]. In contrast, no direct experimental evidence exists for G protein signaling downstream of insect ORs. Several G alpha subunits, in particular G_α_q, are expressed in insect antennae [70–72], but they are not specifically enriched in the ciliated dendrite of OSNs. Reduction of G_α_q levels in *Drosophila* OSNs produces defective behavioral responses to some odor stimuli [73], although it is not known whether this is due to a primary defect in olfactory signal transduction. Surprisingly, OR/OR83b odor-evoked signaling is observed in heterologous cells with or without co-expression of exogenous insect G_α_q proteins [28,29], suggesting that these proteins have the capacity to couple to endogenous, but unknown, signaling molecules. Thus, despite the widespread assumption that insect chemoreception employs a canonical G protein–signaling cascade, the evidence in support of this is inconclusive.

While defining the molecular nature of insect olfactory signaling remains an important goal, our observation that insect ORs are structurally unrelated to the GPCR superfamily raises two equally intriguing possibilities. Insect chemosensory receptors may represent a second family of polytopic TM proteins that has evolved independently to couple to G proteins. Alternatively, these receptors may not couple to G proteins but activate a distinct signaling cascade in response to odor stimulation.

### Implications of This Work and Concluding Remarks

While ORs in mammals and insects share a common function in translating odor stimuli into neuronal activity, our findings reveal fundamental differences in the molecular basis of olfactory perception in these organisms. That their OR families should have unrelated evolutionary origins highlights the remarkable convergence in the anatomical and physiological mechanisms that mammals and insects display in the representation of odors in their peripheral circuits. This work raises important questions about the mechanism of odor recognition and olfactory signal transduction in insects. Furthermore, our demonstration that the OR/OR83b complex is the essential molecular unit of olfactory perception in insects makes this complex an attractive target for the development of highly selective insect repellents to interrupt chemosensory-driven, host-seeking behaviors of insect vectors of human disease.

## Materials and Methods

### *Drosophila* stocks

Fly stocks were maintained on conventional cornmeal-agar-molasses medium under a 12-h light–12-h dark cycle at 18 °C or 25 °C. Mutant alleles and transgenic lines used: *Or83b^1^, Or83b^2^* [22], *Or22a/b^Δhalo^* [7], *Or83b-Gal4, UAS-G-CaMP 1.3* [12], *Or22a-Gal4, Or47b-Gal4* [3], *Gr21a-Gal4* [38], *oseg2-Gal4* [74], *AB1-Gal4* (salivary gland driver) (Bloomington Stock Center, Bloomington, Indiana, United States), *tubP-Gal80ts* [36], *UAS-mCD8:GFP* [75], *UAS-GFP:Rab7* [76]. Genotypes are listed in the figure legends.

### Generation of tagged OR transgenes

All plasmid constructs were generated by amplification of the desired cDNA fragments with flanking restriction sites by PCR from antennal cDNA or cDNA clones, which were T:A cloned into pGEM-T Easy (Promega, Madison, Wisconsin, United States) or pCRII-TOPO (Invitrogen, Carlsbad, California, United States), sequenced and subcloned into appropriate vectors. Tagged OR constructs for transgenic expression were cloned into pUAST [77]. For *Or83b,* we used the ORF and 3′UTR corresponding to nucleotide (nt) 168-1917, of Genbank accession AY567998 in which a previously noted C595T polymorphism was reverted [22]. For *Or43a, Or47b,* and *Gr21a,* we used full-length ORFs only. Epitope tags were fused upstream and in-frame with OR/GR sequences. The GFP tag comprised full-length EGFP (Clontech, Palo Alto, California, United States) lacking the STOP codon. The Rho tag comprised nt 6–65 of Genbank accession M12689, which encodes the antigenically favorable N-terminus of bovine rhodopsin. The *Or83b*:*Or43a* chimera was generated by overlap-extension PCR to avoid introducing restriction enzyme sites at junctions between OR sequences. For the PCA, we used complementary N- and C-terminal fragments of YFP, YFP(1), and YFP(2) with a ten-amino-acid linker [( GGGGS)_2_] [78]. The zipper dimerization domain comprises nt 703–843 of Genbank accession AJ585702, encoding the leucine zipper of *S. cerevisiae* Gcn4p. The artificial signal sequence comprised nt 34–87 of Genbank accession BC002500, which encodes the signal sequence of *Homo sapiens* Calreticulin. Details of all cloning strategies and plasmid sequences are available by request. Transgenic animals were generated (Genetic Services, Cambridge, Massachusetts, United States) and balanced by standard methods.

### Histology

*Antennae:* 14-μm frozen sections of antennae were collected and stained with primary and secondary antibodies (see below) as described [22]. *Salivary glands:* the anterior quarter of the third instar larvae was separated from the rest of the body, inverted to expose the salivary glands, and fixed in 4% formaldehyde in PBS for 20 min. These were then stained as for antennal sections, either in the presence of 0.25% Triton X-100 (Fisher Scientific, Springfield, New Jersey, United States) (permeabilized) or without detergent (non-permeabilized). After mounting in Vectashield (Vector Labs, Burlingame, California, United States), salivary glands were dissected away from other tissues. Images were collected with a Zeiss LSM510 confocal microscope (Zeiss, Oberkochen, Germany).

*Primary antibodies:* mouse α-*Drosophila* Golgi 1:250 (Calbiochem, San Diego, California, United States), mouse α-KDEL 1:100 (Stressgen Biotechnologies, Victoria, British Columbia, Canada), rabbit α-OR83b (EC2) 1:5000 [22], mouse α-OR83b (IC2) 1:100 (University of Texas Southwestern Medical Center Program for Genomic Applications, Dallas, Texas, United States), rabbit α-OR22a/b 1:1000 [22], rabbit α-GFP 1:1000 (Molecular Probes, Eugene, Oregon, United States), mouse α-GFP 1:500 (Molecular Probes). OR83b-specific rabbit polyclonal antibodies against synthetic peptides SMQPSKYTGLVAD (N-term) and HWYDGSEEAKT (IC3) were raised and affinity-purified by Bethyl Laboratories (Montgomery, Texas, United States) and used at 1:1000 and 1:500, respectively. *Secondary antibodies:* Alexa488-, Cy5- and Cy3-conjugated α-mouse IgG or α-rabbit IgG 1:1000 (Molecular Probes; Jackson ImmunoResearch, West Grove, Pennsylvania, United States). DNA was visualized by DAPI staining.

### Optical imaging

In vivo preparation of flies (3- to 8-d-old animals) and optical imaging of odor-evoked calcium responses in the antennal lobe were essentially as described [79] using a modified TILL Photonics imaging system (TILL Photonics, Ludwig Maximilians University, Munich, Germany). For each measurement, a series of 40 frames was taken at 4 Hz. A constant air stream (2,000 ml/min) produced by an aquarium pump was guided through a 1-ml syringe with the tip placed 1 cm from the antennae. Pure odorants were diluted in paraffin oil (see figure legends for concentrations), and 10 μl of this solution was placed on a filter paper in a second syringe that was laterally inserted into this air flow. Odorant stimuli (1 s, i.e., frames 12–16) were puffed into the constant airstream using a custom-made electronic valve under computer control with a 1-min interstimulus interval. Imaging data were analyzed with custom-written IDL software (Research Systems, Boulder, Colorado, United States) including noise filtering, movement, and bleaching correction. Relative fluorescence changes (ΔF/F) were calculated by subtracting the averaged fluorescence intensities from frames 4–6 from the time traces. False-color–coded images (Figures 6A and 7F) represent the signal increase between frames 5 and 16. For traces, 3 × 3 pixel squares were placed onto the center of the relevant glomerulus (marked in the figures) and their values averaged and plotted against time. For each fly we calculated the response to a specific odor as the maximum of frames 12 to 21. The data in Figure 6 were normalized to compare different animals by setting the response to 5% CO_2_ at 100% and scaling the other responses accordingly. Odorants were obtained from Sigma-Aldrich (St. Louis, Missouri, United States) and were of the highest purity available. See figure legends for CAS Registry Numbers. Certified grade CO_2_, diluted to 5% in air, was obtained from Matheson Tri-Gas (Austin, Texas, United States).

### Computational analysis of OR sequences

Multiprotein alignments were generated with ClustalX version 1.83 (Bioinformatics, Strasbourg, France) with default parameters [80]. An unrooted neighbor-joining tree with 1,000 bootstrap replications was generated in ClustalX, excluding positions with gaps and correcting for multiple substitutions. The tree was viewed with TreeView version 1.6.6 (Taxonomy and Systematics at Glasgow, Glasgow, Scotland, United Kingdom) (http://taxonomy.zoology.gla.ac.uk/rod/rod.html). The sequences analyzed here are divergent and share extremely low homology between protein families, and the intent of this analysis was to ask whether *Drosophila* ORs cluster with olfactory GPCRs. Membrane topology predictions of OR sequences were examined using HMMTOP version 2.0 [48], TMHMM Server version 2.0 [49], and transmembrane prediction [50]. The set of 61 mouse ORs was chosen to maximize representation of subfamily members. N-glycosylation sites were predicted using the NetNGlyc 1.0 Server (http://www.cbs.dtu.dk/services/NetNGlyc). Graphical representations of OR sequences as snake plots were adapted from Residue-based Diagram editor outputs obtained from the GPCR database [81]. The accession numbers of all protein sequences analyzed are available on request.

### Topology-mapping with β-gal fusion proteins

*lacZ* and *TM:lacZ* (encoding β-gal with an upstream synthetic TM domain) were amplified from pPD16.43 and pPD34.110 [82], respectively, and cloned downstream of *OR* and *Rh1* cDNA fragments in pMT-V5-His A (Invitrogen). Codons of test genes fused to *lacZ* and *TM:lacZ:* OR83b (1–46, 1–72), OR9a (1–41, 1–68), RH1 (1–49, 1–82). Plasmids were transfected into S2-R+ cultured *Drosophila* cells [83] with Fugene (Qiagen, Valencia, California, United States). Expression of fusion proteins was induced for 24 h with 5 mM Cu_2_S0_4_ and verified by Western blotting of cell extracts using antibodies against β-gal (Cappel from MP Biomedicals, Irvine, California, United States). To assess β-gal activity, cells were rinsed in PBS, fixed in 2% formaldehyde/0.2% glutaraldehyde in PBS for 5 min, rinsed twice in PBS, and incubated in staining solution [5 mM K_3_Fe(CN)_6_, 5 mM K_4_Fe(CN)_6_.6H_2_0, 2 mM MgCl_2_, 1 mg/ml X-gal, in PBS] for 30 min at 37 °C. After staining, cells were rinsed in PBS and viewed on an inverted microscope.

### Electron microscopy

*Conventional transmission electron microscopy:* flies were immobilized on ice and transferred to ice-cold 2.5% glutaraldehyde in 0.1 M cacodylate (pH 7.4). Antennae were fixed overnight in the cold, rinsed with buffer, and post-fixed with 1% osmium tetroxide in 0.1 M cacodylate (pH 7.4) on ice. After en bloc staining with aqueous uranyl acetate, the tissue was dehydrated in a graded alcohol series and embedded in hard-grade L.R. White (Electron Microscopy Sciences, Hatfield, Pennsylvania, United States).

*Prelabeling immunoelectron microscopy:* 6-μm cryostat sections of fresh-frozen adult antennae were collected on an Aclar film (EMS) treated with Alcian blue. Sections were fixed for 10 min in 4% paraformaldehyde, washed in PBS, and incubated with α-EC2 (diluted 1:5,000) overnight in the cold. After washing with PBS, the sections were incubated with goat-α-rabbit conjugated to 5 nm gold particles (Amersham Biosciences, Little Chalfont, United Kingdom). Incubations were stopped by buffer washes followed by fixation with 2.5% glutaraldehyde. Sections were further processed as described above except embedding was in EPON. Silver sections were collected on copper grids and viewed unstained in a JEOL 100 CX electron microscope operated at 80 kV. Distributions of gold particles were scored blindly by an impartial observer.

### Yeast two-hybrid analysis

*OR*/*GR* fragments were cloned into GAL4 DNA-binding domain or activation domain vectors pGBK-T7 and pGAD-T7 (Clontech). OR fragments used (amino acid codon number): OR83b N-term (1–47), OR83b IC1 (97–134), OR83b IC2 (226–351), OR83b IC3 (412–459), OR43a IC2 (208–246), OR43a IC3 (298–342), OR22a IC3 (305–359), GR21a IC3 (357–416). These were transformed into yeast strain AH109 (Clontech) using standard procedures, and expression of fusion proteins was verified by Western analysis using antibodies against GAL4 (Clontech). Interactions were tested by restreaking six single cotransformant colonies on media selecting for expression of the *HIS3* and *ADE2* reporter genes and scoring for growth after 3 d at 30 °C.

## Supporting Information

### Accession Numbers

The accession numbers of all protein sequences analyzed are available on request.

## Abbreviations

EM: electron microscopy
ER: endoplasmic reticulum
GFP: green fluorescent protein
GPCR: G protein–coupled receptor
GR: gustatory receptor
nt: nucleotide
OR: odorant receptor
OSN: olfactory sensory neuron
PCA: protein-fragment complementation assay
RH1: rhodopsin 1
TARGET: temporal and regional gene expression targeting
TM: transmembrane
YFP: yellow fluorescent protein
